# Efficient vasculature investment in tissues can be determined without global information

**DOI:** 10.1098/rsif.2020.0137

**Published:** 2020-04-22

**Authors:** Salva Duran-Nebreda, Iain G. Johnston, George W. Bassel

**Affiliations:** 1 School of Life Sciences, University of Warwick, Coventry CV4 7AL, UK; 2 Faculty of Mathematics and Natural Sciences, University of Bergen, Bergen, Norway

**Keywords:** generative model, vasculature, dimensionality, network, spatial constraint, organ design

## Abstract

Cells are the fundamental building blocks of organs and tissues. Information and mass flow through cellular contacts in these structures is vital for the orchestration of organ function. Constraints imposed by packing and cell immobility limit intercellular communication, particularly as organs and organisms scale up to greater sizes. In order to transcend transport limitations, delivery systems including vascular and respiratory systems evolved to facilitate the movement of matter and information. The construction of these delivery systems has an associated cost, as vascular elements do not perform the metabolic functions of the organs they are part of. This study investigates a fundamental trade-off in vascularization in multicellular tissues: the reduction of path lengths for communication versus the cost associated with producing vasculature. Biologically realistic generative models, using multicellular templates of different dimensionalities, revealed a limited advantage to the vascularization of two-dimensional tissues. Strikingly, scale-free improvements in transport efficiency can be achieved even in the absence of global knowledge of tissue organization. A point of diminishing returns in the investment of additional vascular tissue to the increased reduction of path length in 2.5- and three-dimensional tissues was identified. Applying this theory to experimentally determined biological tissue structures, we show the possibility of a co-dependency between the method used to limit path length and the organization of cells it acts upon. These results provide insight as to why tissues are or are not vascularized in nature, the robustness of developmental generative mechanisms and the extent to which vasculature is advantageous in the support of organ function.

## 1. Introduction

Multicellularity has evolved independently multiple times during the evolution of our biosphere [1]. Extant multicellular organisms span several orders of magnitude in size [2,3]. Increased size has been suggested to allow organisms to inhabit new niches and thus ensure limited competition and enhance their survival [4]. However, increased size incurs additional challenges to the functioning of organs and organisms [5], such as the effective distribution of resources across the multicellular entity as well as coordination of biological action via information transfer [6]. These challenges include responses to changing environments and the timing of developmental transitions [7,8].

To tackle these issues, specific transport-oriented cell types and systems have evolved, including respiratory and vascular systems [9–12]. Vascular elements create a delivery system that effectively reduces the scale of the tissues and organs they are part of, introducing fast routes to transfer information and matter across cells in tissues [13,14]. This allows larger organs to be less constrained by their dimensionality (whether they are two dimensional or three dimensional) and scale [5,15].

Vasculature is, however, a costly investment in the physiology of organisms [10], as it is dedicated to supporting the delivery of nutrients and information to other cell types, rather than supporting organ function directly [3]. As a result, vascular elements need to be efficiently built and managed [16]. In this study, we investigate the extent to which a multicellular system can be effectively vascularized without coming at a prohibitive cost to organ function.

The relevance and ubiquity of delivery networks in biology have prompted the search for fundamental mathematical principles that pervade transport networks [3,14,16–18]. It has long been identified that transport systems often display allometric scaling [17,19,20], with power laws connecting biological variables with reasonably well-defined exponents [3,19–21]. These have been shown to operate in multiple biological substrates, such as animal respiratory systems [20,22,23], cardio-vascular systems [20,24], tumours [25,26], plant transport networks [27–29] and even foraging trails of ants [30]. Theories have been proposed to explain such mathematical regularities, including the hierarchical and symmetric branching of vessels in the transport network [3,18,31]. This approach is able to predict, in an already established network, the average properties of vessel radius and vessel length changes across the system.

A different approach considers how this transport system might be dynamically generated, an aspect of key importance in understanding the development and regeneration of such systems. For example, optimality has been explored in ‘top-down' vascular networks which begin as fully connected uniform systems that optimize flow and particle delivery [32]. This process has been argued to closely replicate the biological processes behind retina development [33]. This type of model has also provided insights into organ regeneration by exploring the relation between damage and structure [34]. In this article, we take an inverse perspective, and explore biologically plausible ‘bottom-up' generative models of vascular development [35]. This allows us to consider, from first principles, the fundamental rules of a generative process capable of creating shortcuts in a tissue in order to reduce the impact of scale. Using a dynamic network science framework, we investigate the extent to which a multicellular system can be effectively vascularized without coming at a cost to organ function, as vascular cells provide transport and support to organs but do not contribute to the biochemical or physiological function that the organ performs.

## 2. Results

### 2.1. A general model to simulate vascular development in cell contact networks

In order to investigate the dynamics of vasculature creation in a model tissue network, we propose a generative process based on node centrality computation and node fusion. In this model, nodes of the network stand for cells and edges represent cellular physical contacts. Lattices were used to represent immobilized cells within tissues, as observed in spatially constrained organs, with typically homogeneous connectomes [36]. The premise is that vasculature formation is equivalent to the strings of cells being fused into a single node, which reduces network distances within the graph [6,37], and that only contiguous cells can be fused together in this process. This process would be analogous to the formation of a cavity in animal vascular systems, where solutes and cells in suspension can move freely inside vascular vessels. But this system is also similar to vasculature formation in plants, where the individual cells of the phloem are connected end-to-end through the sieve plate, which contains enlarged holes that connect contiguous cells together in a single cytoplasm. For the sake of simplicity, we consider only undirected and unweighted graphs in this approach.

Node centrality computation informs the algorithm about which nodes have a greater impact in the relaying of information within the network (see Methods). The physical limitation of information transfer and distances in a tissue is related to the feasibility of coordinating developmental transitions as well as coordinating responses to environmental changes or biological damage [6,7]. Node fusion reduces distances in the network and facilitates information transfer by creating shortcuts. To quantify these effects, we consider a read-out of our generative model, namely the average path length of the network after successive iterations of the algorithm [38]. Average path length is a biologically relevant read-out metric as it captures distances between sources of information and their destination across the tissue where both source and receptor location are not known *a priori*.

**Figure 1.**
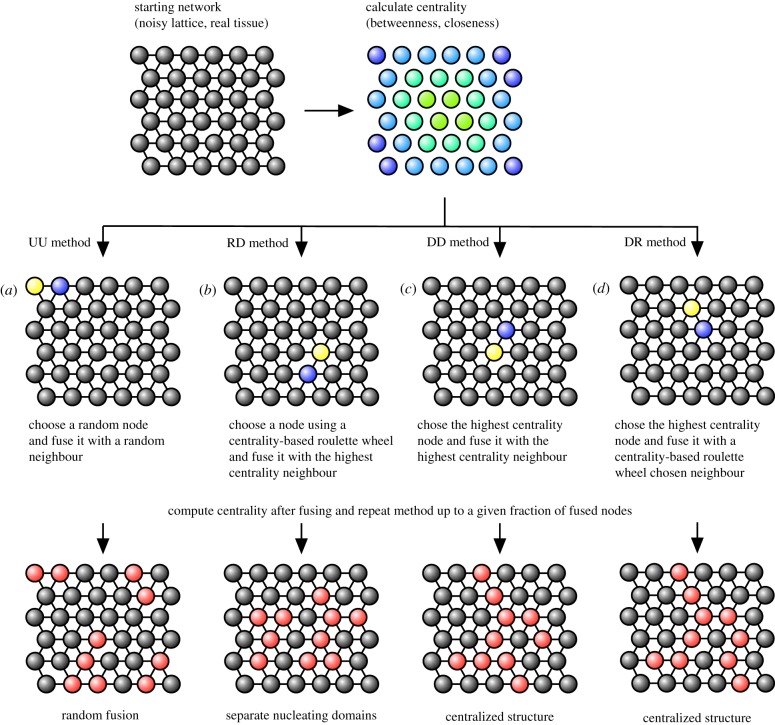
Schematic representation of the algorithm used in this article. The starting network (top) is a real epithelial network or a noisy regular lattice in two, three or 2.5 dimensions (three layers of hexagonal lattice where the vascular elements can only propagate though the middle layer). In this graph, node centrality is calculated, either as closeness centrality or betweenness centrality. Then, a node from the network is chosen (blue node) and a first-order neighbour of this node is determined (yellow node) using one of the four sets of rules described in the middle row. The nomenclature shown represents whether the algorithm used to choose the first and the second nodes follows a deterministic (D) rule that identifies the global highest centrality node or a random (R) rule that makes a probabilistic selection over high-centrality nodes. When centrality does not inform node choice, the resulting purely random process is labelled UU, for the uniform random choice in both stages. These two nodes are fused (red nodes), generating a new graph that is fed again into the algorithm. Finally, after a given fraction of cells have been turned into vascular elements, the algorithm ends.

The proposed algorithm operates with the following steps (figure 1):

(i) compute node centrality for each vertex in the graph;
(ii) choose a node based on the centrality values;
(iii) choose a first-order neighbour of the node chosen in (ii) based on the centrality values;
(iv) fuse the nodes chosen in steps (ii) and (iii);
(v) go to step (i).

Different methods are used to select which nodes are fused in each iteration (figure 1). A node can be chosen at random and a random first-order neighbour to be fused with it (method UU for uniform, figure 1*a*). An alternative method considers choosing the specific, globally identified node with the highest centrality and its highest centrality neighbour (method DD for deterministic–deterministic, figure 1*b*). A third method uses a roulette wheel algorithm [39] with propensities proportional to the node centrality for selecting the first node (hence not guaranteeing that the global maximum is identified) and the highest centrality for the neighbour node (RD method for random–deterministic, figure 1*c*). The fourth method is the reverse of the previous method, whereby the globally highest centrality node is chosen but a roulette wheel algorithm with centrality-based propensities is then used in order to choose the neighbour to be fused to (DR method for deterministic–random, figure 1*d*). This method results in the growth of the vasculature from the ends of existing vascular elements.

Upon fusion, a new graph is obtained, and the centrality is computed again in this reduced network. Average path length is also computed and the trade-off between vasculature investment and path length reduction is explored.

We choose network centralities as the governing feature of cells for vascular selection because these measures have been shown to correlate with cell behaviour in three-dimensional (3D) organs [40]. While cells may not be able to directly compute how central they are in terms of the network theory methods discussed here, centralities are known to positively correlate with easily sensed physiological variables; hence, they provide roughly actionable quantities in tissues [40] and are suitable substitutes for biologically driven processes [41,42].

**Figure 2.**
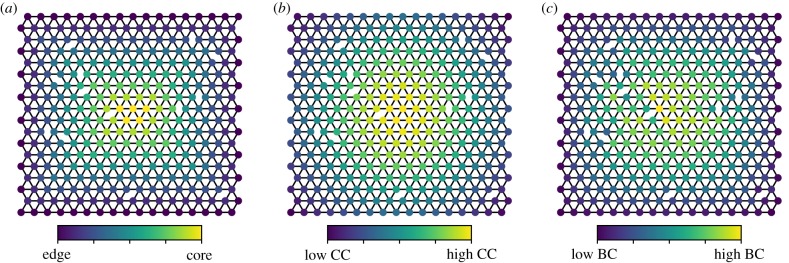
Centrality computation in a 2D hexagonal lattice with 300 nodes. (*a*) Distance to the boundaries of the graph (here determined by the position of the nodes). (*b*) Betweenness centrality, and (*c*) closeness centrality. Betweenness and closeness differ in the impact that the random removal of edges has in the centrality distribution. CC maps more closely to the distribution found in (*a*), which may represent an external gradient established by diffusion from the outside of the tissue towards the centre [44].

More specifically, we considered two centrality measures linked to the physical structure of tissues. First, closeness centrality (CC) measures the mean distance to all other nodes in a network [43]. In the case of a lattice, this also correlates with the distance to the network boundary (figure 2*a*,*b*). This maps reasonably well to how cells are positioned within an organ. The plausibility of CC as an actionable stimulus is supported, for example, by a study in plants showing that cells can sense their embeddedness within a plant organ following an oxygen gradient [44]. In this case, the stability of a transcription factor was shown to be linked to oxygen concentration, which in turn drives spatially derived gene expression patterns. Cells may therefore sense how deep they are within a tissue using such external gradients, demonstrating that the physical distance from the boundary of the organ can play an instructive role in cell behaviour.

Second, we consider betweenness centrality (BC), which uses the knowledge of a complete network to identify nodes that lie upon the greatest number of shortest paths [45]. Such nodes are poised to mediate the greatest amount of system-level information flux. Like CC, BC also maps to nodes in the central region of a lattice (figure 2*c*) owing to the fact that these inner cells are mediating more information transfer. While BC is probably not computed directly by a tissue (as it requires a global understanding of cellular organization), other methods which only make use of local information, such as navigation centrality (NC), which identifies shortest paths by following gradients, correlate strongly with BC [46], providing a link between the biologically plausible method of NC and BC. Additionally, in the electronic supplementary material, we show that BC positively correlates with the flux of diffusible toxic byproducts simulated on a two-dimensional (2D) template, further validating the use of BC as a computationally tractable stand-in of purely local information processes. We also note that BC is linked with readily sensed physical quantities, including surface area of a cell and its volume [40]. Owing to the high computational cost of computing NC and physiological processes, as well as the comparable behaviour to BC, we make use of BC in this study.

The centrality quantity that is used is incorporated in the naming conventions as a subscript: either BC for betweenness centrality or CC for closeness centrality.

### 2.2. Vasculature performance in 2D sheets of cells

**Figure 3.**
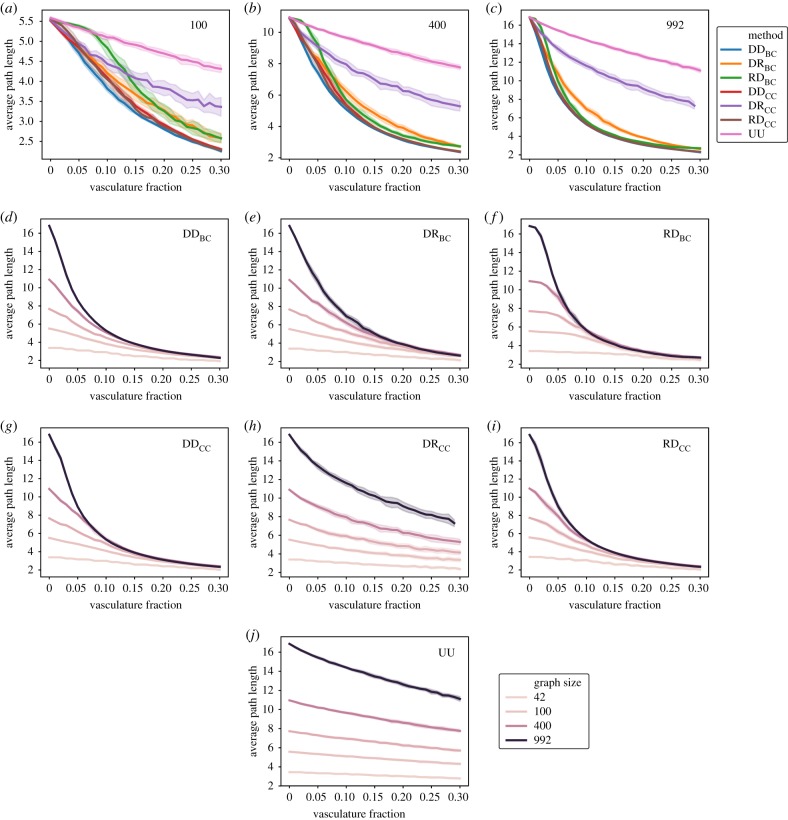
Average path length scaling for an increasing fraction of cells converted to vasculature in noisy 2D hexagonal lattices. Comparison between the different methods described in the Methods section (*a*–*c*). For graphs of constant size (*a*) 100, (*b*) 400 and (*c*) 992, the effect of node fusion following the high betweenness (DD_BC_), roulette wheel then highest value betweenness (RD_BC_), highest value then roulette wheel betweenness (DR_BC_), high closeness (DD_CC_), roulette wheel then highest value closeness (RD_CC_), highest value then roulette wheel closeness (DR_CC_) and random methods (UU). Scaling for each of the methods in different graph sizes (*d*–*j*). Exploration of the methods DD_BC_ (*d*), DR_BC_ (*e*), RD_BC_ (*f*), DD_CC_ (*g*), DR_CC_ (*h*), RD_CC_ (*i*) and R (*j*) in graphs ranging from 42 to 992 nodes. All data points contain 25 replicates; shaded regions represent a standard deviation of the sample.

The average path length for each vascularization method decreases with the fraction of vascularization, as might be expected (figure 3). The random method (figure 3*a–c*,*j*) performs very poorly when compared with all other methods, only achieving a 26% reduction in average path length when 30% of the cells of 992 node networks have been fused. The DR_CC_ method (figure 3*a*–*c*,*h*) also performs weakly, achieving at best a 50% reduction in the same conditions. The remaining methods, strikingly, behave quite similarly, all inducing a dramatic improvement in path length with vascular fraction in the 992 node tissues. Notably, methods leveraging global knowledge such as DD_BC_ do not dramatically outperform more local information methods such as the roulette approach of RD_BC_ or DR_BC_. This separation of behaviours in three groups is lost as the size of the networks is decreased, with similar behaviours obtained in the DR_CC_ and the rest of the non-random methods. It is particularly interesting to note that the RD_BC_ method displays two regimes at the lower end of graph sizes, one where it performs as poorly as the random method and a second regime, as the fraction of fused cells increases, where it becomes as efficient as the DR_BC_ method.

A fundamental difference between the path length reduction of DD_BC_ and RD_BC_ can be observed in figure 3*d*,*f*. In the case of DD_BC_, different graph sizes show different trajectories as the vasculature fraction is increased. In the case of RD_BC_, the starting point is a function of the graph size, but different graph sizes more readily coalesce into a single trajectory as the vasculature fraction is increased. This overlap means that the average path length becomes a function of vasculature fraction alone and is independent of the scale of the template. While the RD_BC_ approach may lack tangibility in terms of the observed creation of continuous and coherent vascular systems (see below), this suggests a different class of underlying mechanics of how these generative processes could give rise to vascular systems which transcend path length. Importantly, different vasculature generative mechanisms provide scale-free improvements in transport, pointing to a robust biological mechanism to establish these systems.

### 2.3. Vasculature performance in 3D assemblies of cells

**Figure 4.**
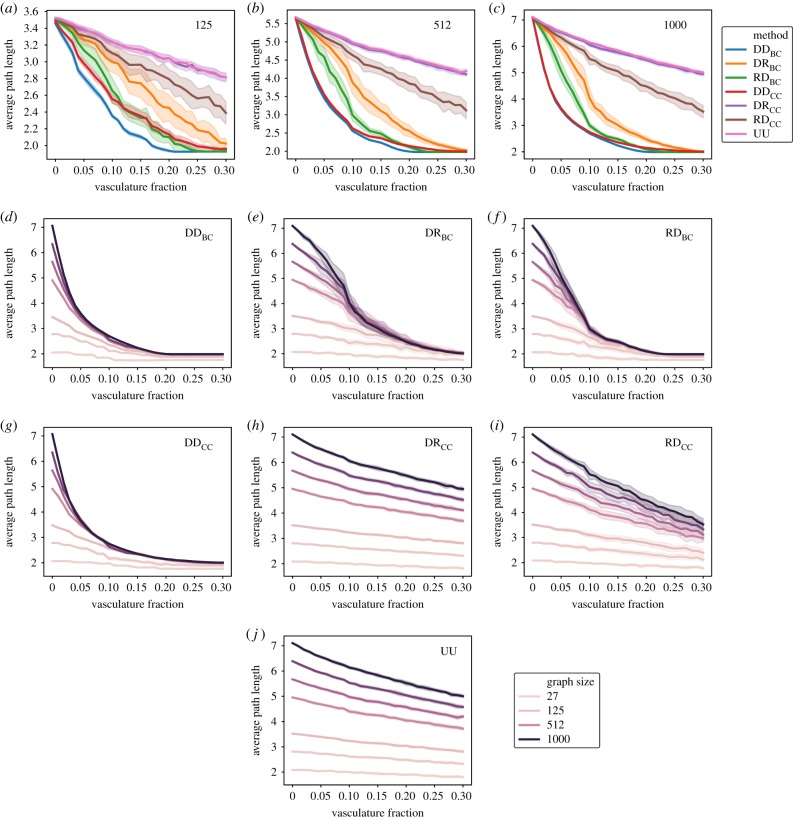
Average path length scaling for an increasing fraction of cells converted to vasculature in noisy 3D hexagonal lattices. Comparison between the different methods described in the Methods section (*a*–*c*). For graphs of constant size (*a*) 125, (*b*) 512 and (*c*) 1000, we show the effect of node fusion following the high betweenness (DD_BC_), roulette wheel then highest value betweenness (RD_BC_), highest value then roulette wheel betweenness (DR_BC_), high closeness (DD_CC_), roulette wheel then highest value closeness (RD_CC_), highest value then roulette wheel closeness (DR_CC_) and random methods (UU). Scaling for each of the methods in different graph sizes (*d*–*j*). Exploration of the methods (*d*) DD_BC_, (*e*) DR_BC_, (*f*) RD_BC_, (*g*) DD_CC_, (*h*) DR_CC_, (*i*) RD_CC_ and (*j*) R in graphs ranging from 27 to 1000 nodes. All data points contain 25 replicates; shaded regions represent a standard deviation of the sample.

Figure 4 shows the same analysis discussed in figure 3 for 3D networks instead of 2D. In 3D tissues, roulette wheel methods show a higher spread across replicates than in 2D tissues, while non-stochastic methods such as DD_CC_ and DD_BC_ follow a deterministic trend that only contains the stochasticity included during the network creation. Similarly to overall trends observed at two dimensions in smaller sized graphs, the performance of different methods overlaps in 125 node graphs (figure 4*a*) compared with 1000 node graphs (figure 4*c*). Independently of graph size, the DR_CC_ method performs particularly poorly in 3D networks (figure 4*h*), comparable to the random method (figure 4*j*). In contrast with the 2D lattice case, the RD_CC_ method now also performs poorly (figure 4*i*). A lag in path length reduction and later improvement is also observed here in the RD_BC_ method (figure 4*f*), a consequence of percolation transition taking place as the number of fused nodes increases [47].

Interestingly, the gap between full information methods (DD_BC_, DD_CC_) and local information methods (RD_BC_, DR_BC_, RD_CC_, DR_CC_) is widened with respect to the 2D template discussed before. While in two dimensions, there is an overlap and all these methods performed very similarly at the intermediate vascular fractions (about 15%), in the 3D models a wider set of behaviours is observed at this stage. However, performance compared with the best methods recovers as the vasculature fraction increases, reaching similar levels of path length reduction at the 30% vasculature fraction mark.

### 2.4. Vasculature performance in 2.5D tissues

**Figure 5.**
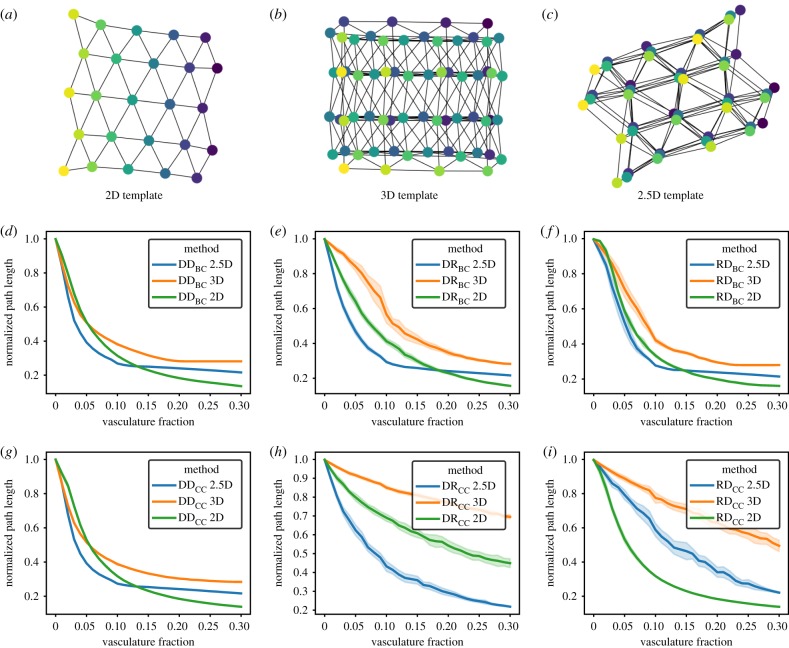
Algorithm operation in constrained 3D systems similar to plant leaves, which we refer to as 2.5D. As a visual guide, we show small examples of the three types of synthetic templates discussed so far: (*a*) two dimensions, (*b*) three dimensions and (*c*) 2.5 dimensions. Node colour corresponds to the depth dimension, which cannot be visualized in a 2D representation of this 3D layout. For systems of 18 × 18 × 3 nodes (2.5 dimensions), 1000 nodes (three dimensions) or 992 nodes (two dimensions), we show the normalized average path length reduction as the fraction of cells transformed into vasculature is increased. For comparison, the equivalent experiments in two and three dimensions are shown. Methods include (*d*) DD_BC_, (*e*) DR_BC_, (*f*) RD_BC_, (*g*) DD_CC_, (*h*) DR_CC_, and (*i*) RD_CC_. All data points contain 25 replicates; shaded regions represent a standard deviation of the sample.

Many tissues have a layered organization with vascular elements existing solely within their inner structures. The leaf of a plant is one such example, having distinct non-vascularized epidermal tissues and a vascular system within. Here we refer to these structures as a constrained 2.5-dimensional (2.5D) system (between two and three dimensions with respect to vascular system generation). These networks were built using a 3D hexagonal template but restricting one of the dimensions to three rows of cells. 2.5D lattices (figure 5*c*) are a useful system to investigate how vasculature growth might operate in leaves and other tissues, which are strictly 3D, but where one of the dimensions of the system is actually not as equally extended as the remaining ones.

Figure 5*d*–*i* shows the comparison in normalized path length reduction in 2D, 3D and 2.5D tissues for the methods discussed previously. Except in the case of DR_CC_ methods, there is a consistent endpoint structure to the normalized path length reduction at maximum investment in vasculature, with the highest path length going to three dimensions, the smallest path length to two dimensions and 2.5 dimensions achieving an intermediate value. Interestingly, in the DD_BC_, RD_BC_, DR_BC_ and DD_CC_ methods, another common feature can be found, with 2.5 dimensions being the best-performing template in terms of normalized path length reduction at lower vasculature fractions. However, these common features are not universal, suggesting that there is not a single algorithm that performs best in all tested substrates [48].

### 2.5. Network analysis of vascular systems

**Figure 6.**
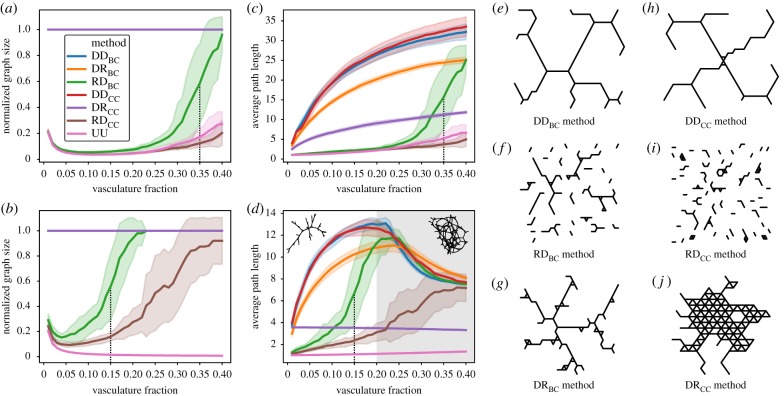
Effects of percolation dynamics as the fraction of fused nodes increases. For the largest templates in (*a*) 2D and (*b*) 3D graphs, we characterize the average graph size (nodes included in each vascular element) normalized by the maximum sized graph that could be obtained at that particular vasculature fraction. We also show for (*c*) two dimensions and (*d*) three dimensions the average path length within elements of the vascular system. As an inset in (*d*) an example of 3D vasculature network at 0.15 (left) and 0.3 (right) is shown, using the RD_BC_ method on a 1000 node 3D synthetic template. All data points contain 25 replicates; shaded regions represent a standard deviation of the sample. (*a*–*d*) We show the transition point for the RD_BC_ method as a vertical dotted line, where the curvature switches from positive to negative. We also provide, as a grey-shaded region, the domain of path length reduction for 3D methods in (*d*). For the different methods used in this study: (*e*) DD_BC_, (*f*) RD_BC_, (*g*) DR_BC_, (*h*) DD_CC_, (*i*) RD_CC_ and (*j*) DR_CC_, we show a representative simulation of the vascular system created in a 0.1 vasculature fraction in a 992 node 2D synthetic template. Average path length in disconnected sets of vascular vessels is computed as the average path length of the largest component in terms of the number of nodes.

Methods that use the roulette wheel algorithm for selecting the first node in the fusion step (UU and RD) typically initially create disconnected elements of vasculature. Over time, these disconnected components then come together in a single element, a phenomenon described as the percolation transition [47,49–52]. Before this transition happens, RD_BC_ behaves similarly to the random, uninformed method. However, as the number of nodes fused increases, RD_BC_ can perform almost as well as the equivalent methods requiring global information (DD_BC_). The vertical dashed lines in figure 6*a*,*c* show these transitions happening in two and three dimensions, respectively. In two dimensions, roughly 35% of node fusion is needed to obtain a transition in the simulation (dotted line, figure 6*a*). Conversely, this transition happens much earlier in three dimensions, with only 15% of vasculature needed to percolate (figure 6*c*).

A different set of behaviours is observed between two and three dimensions in terms of average path length within the virtual vascular system. In two dimensions, the DD_BC_, DR_BC_, DD_CC_ and DR_CC_ methods display a logarithmic growth in average path length, while RD_BC_ shows a sharp increase in average path length at the values of vasculature fraction where the transition takes place. Some of the methods that show a logarithmic growth in two dimensions display in three dimensions a decrease in average path length owing to loop formation in the vascular system beyond 20% vasculature investment (figure 6, inset). Figure 6*e*–*j* shows a representative simulation of vasculature systems for each of the 2D methods at 10% vasculature fraction. The fragmented nature of the vascular elements created by RD_BC_ and RD_CC_ before the percolation transition is shown in figure 6*f*,*i*.

### 2.6. Algorithm operation on biological templates

**Figure 7.**
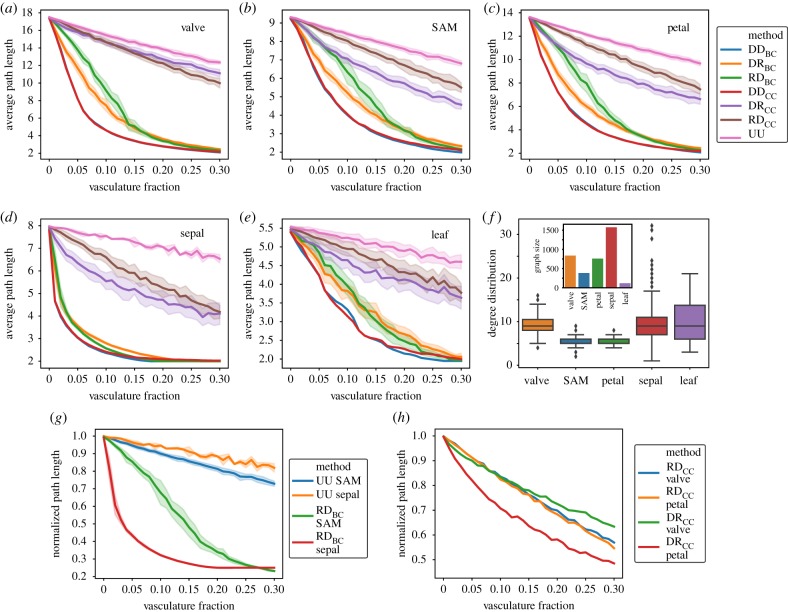
Algorithm operation across real templates of plant epithelial networks (https://osf.io/fzr56/). For (*a*) valve, (*b*) SAM first layer, (*c*) petal adaxial, (*d*) sepal abaxial and (*e*) leaf adaxial epidermal tissues, different methods presented in this study reduce average path length to varying extents as the vasculature fraction increases. All data points contain 25 replicates; shaded regions represent a standard deviation of the sample. In (*f*), we show the degree distributions of all the tissues shown, the box limits represent the quartiles of the data, the solid line inside the box represents the median of the distribution and the whiskers stand for the range of the distribution, with outliers shown as dots. As an inset, the sizes of the template graphs are shown. (*g*,*h*) Comparison between UU and RD_BC_ method performance in SAM and sepal epithelia, as well as RD_CC_ and DR_CC_ in valve and petal epithelia. Average path length shown in (*g*) and (*h*) is normalized by the highest average path length observed in the series, typically corresponding to the value at no vasculature.

Following the observation that no single algorithm performs best on all templates, we sought to evaluate the relationship between these vasculature generative methods and the different templates upon which they act. To this end, we made use of biological cellular connectivity data collected from different organs of the plant *Arabidopsis thaliana*. We focused on the epidermal cell layers to control for the topological diversity that is present across the 3D organs of the plant (https://osf.io/fzr56/) [40]. These templates do not contain vascular cells themselves, as vascular cells are extremely difficult to capture reliably using confocal microscopy for all tissues and are thus removed from all the organs used in this model. We studied the relationship between path length and vascularization for epidermal tissues, including the valve epithelia, the shoot apical meristem (SAM) first layer, the petal cone cell epithelia, the sepal abaxial epithelia and the leaf adaxial epithelia (figure 7*a*–*e*). Differences in algorithm performance across the templates can be readily observed, a particularly notable example being the comparison between the poorly performing uniform method (UU) and the roulette-based BC method (RD) in figure 7*g*. Comparing the effects these two methods have in SAM epithelia and sepal epithelia, a reversal in trend can be observed. While in the sepal, the RD_BC_ method rapidly decreases the average path length (more so than the same method in the SAM epithelia), the reverse trend in performance can be observed using the UU method (figure 7*g*).

This suggests that, in order to achieve optimal performance, there should be an interplay between the algorithms and the templates in which they operate. In particular, the sepal epithelia have a much broader degree distribution than the SAM (figure 7*f*), with giant cells that naturally form shortcuts across this template. These higher degree cells (some of them outliers in the distribution shown in figure 7*f*) have a large betweenness centrality and guide the process of vasculature formation for both RD_BC_ and DD_BC_ methods. This effect is shown to be reversed in other methods (figure 7*g*). Another example of this same feature is shown in figure 7*h*, where RD_CC_ and DR_CC_ are tested in valve and petal epithelia. In this example petal and valve show a switch in which method performs better, RD_CC_ or DR_CC_. This may impact the formation of vasculature in different organs depending on the organization of cells and the algorithms invoked in the construction of such systems.

### 2.7. Optimality in vasculature construction

To identify the efficient vascular system construction and reduction of node distances across simulated vascular elements, we examined the extent to which the global transport efficiency of multicellular systems is influenced by different percentages of vascularization. The DD_BC_ and DD_CC_ methods were selected based on their ability to effectively generate coherent vascular systems (figures 3 and 4).

**Figure 8.**
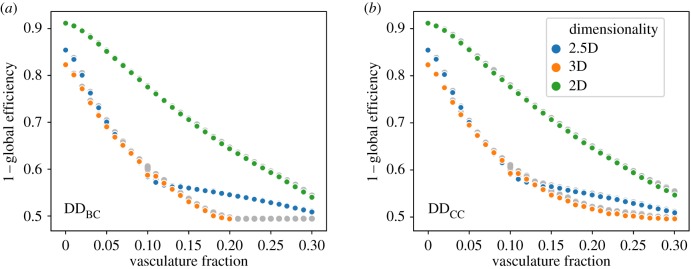
Optimality in vascular systems across template dimensionalities. For the best reported methods (*a*) DD_BC_ and (*b*) DD_CC_, we show the relation between investment in vasculature and global efficiency improvement in the largest templates (972, 992 or 1000 nodes for 2.5, two or three dimensions, respectively). The non-dominated front of solutions is displayed (solid points in colour) as well as dominated solutions (grey, semi-transparent points), following the minimization of both objectives (vasculature fraction and 1 – global efficiency). The results for the three synthetic classes of templates, two, three and 2.5 dimensions, are shown independently.

Figure 8 shows trade-offs [53] describing the best observed solutions when presented with vascularization as a multi-objective problem, trying to obtain the best global efficiency with the least investment in vasculature. A clear difference in how these algorithms operate in two dimensions versus 2.5 and three dimensions is observed. The 2D process results in a progressive lowering of path length. By contrast, clear transitions of diminishing returns are present in 2.5D and 3D templates, and at a lower relative threshold in the former than in the latter. While these thresholds are dependent upon the size of the network, the presence of these transitions in higher dimensional templates indicates that optimal thresholds of vascularization are present.

A ready connection of the trade-off shown here can be made to the process of pruning after the establishment of the primitive vascular plexus [54]. In the biological construction of vasculature during skin development, an over-connected mesh of vascular vessels is formed (high vascular fraction with low path length) that is pruned efficiently, transforming it into a branching tree (lower vascular fraction but higher path length and lower global efficiency).

## 3. Discussion

Here we have explored how a set of algorithms that can build delivery systems in a spatially embedded tissue perform under different conditions, including varying tissue scale, dimensionality and topology, in synthetic and experimentally characterized cellular connectivity networks.

The best performance in path length reduction when comparing different methods is typically found in algorithms that use more information, namely DD_CC_ and DD_BC_, which require non-local information on node centrality in order to choose the direction of growth for the vascular element (figures 3 and 4). However, methods using more local information, simulated here with the use of a roulette wheel algorithm that represents rates of growth in different directions acting independently, can achieve similar levels of success, especially in large graphs and high vasculature fraction conditions. Strikingly, we find that scale-free improvements in transport can be achieved in the absence of global knowledge of the system.

The RD_BC_ method also offers the interesting property of scale invariance, with equal values of average path length with a given vasculature fraction for different network sizes (figures 3 and 4). As different nucleating elements come together to form a single vascular system, a percolation transition takes place, which gives rise to scale-free behaviour. These two findings suggest that less informed systems (local versus non-local information and organ size information) might not be required in order to efficiently construct an effective vascular system.

The effect of different templates on the performance of these algorithms was also investigated. Differences in performance following graph dimensionality (two, 2.5 and three dimensions) were observed. Optimality analysis using a Pareto front exploring the extent of investment in vascularization versus global transport efficiency identified the presence of critical thresholds in 2.5D and 3D networks, beyond which no additional gains are obtained by making more vasculature (figure 8).

Unlike in 2.5D and 3D networks, 2D tissues did not show a rapid decrease in global transport efficiency with increased vasculature, and the Pareto front did not overlap with the other dimensionalities analysed (figure 8). This lack of improvement from vasculature may be one reason why two-dimensional vascularized organs or organisms are not observed in nature.

Further differences in template dimensionality were observed, whereby loops formed in 3D templates using the vasculature generative processes, which were not observed in 2D templates using these same algorithms (figure 6). This in turn impacted the reduction of path length in these transport systems. Loops have also been linked to increased resilience to damage and fluctuations [34].

Finally, we tested the same algorithms in different real 2D templates obtained from plant epidermal layers. This analysis shows that there are trade-offs at play, and that strong improvement for one method in a specific template may come at the cost of performance in other templates (figure 7). This also suggests that adaptation and optimization in vasculature-creating processes can come from the molecular mechanisms that decide the construction (algorithm) as well as the physical substrate organization in which they take place (template). Characterization of tissue templates in terms of size, dimensionality and topology can be informative of the algorithmic processes that might be operating in them.

Altogether these results highlight that optimality in the extent and algorithm used for the vascularization of tissues is dependent on the size, dimensionality and topology of the cellular templates. This study provides a framework to explain optimal organ design [55] within the context of long-distance delivery systems that transcend path length. Application of this knowledge extends to diverse spatially embedded multicellular systems, and the lack of observed vascularization in cellular monolayers. These results, however, rest on the premises of the model. Namely, we did not introduce some well-known mechanical constraints such as flow conservation equations when choosing the next node to be fused. Further expansions of this framework could include flow conservation features to better match observed vasculature bundle width and mechanical properties, as well as allometric scaling in delivery systems as previously defined [3].

## 4. Methods

### 4.1. Template construction and vascularization methods

In this study, we represent multicellular organs as a connectivity network of cells. These template networks correspond to two classes: regular lattices with bounded stochasticity (random deletion of edges to break the symmetry of the lattice, see below) or real tissue networks presented in previous studies about epithelia (https://osf.io/fzr56/). In the case of regular lattices, 2D, 2.5D or 3D triangular lattices were created using the NetworkX library for Python [56]; here, 2.5D refers to an otherwise 3D template being restricted to only three stacked layers (see figure 5 for a clear example of a 2.5D template). First, a regular square lattice was created using standard NetworkX functions. Then, a staggering of node positions in alternating layers was introduced to approximate a triangular lattice. Finally, the network was reconnected using Euclidean distance between pairs of nodes (using the row, column and depth as coordinate systems for the Euclidean system).

In order to introduce variation across each of the independent runs of the model, a constant fraction of edges was deleted in the case of synthetic templates, whether two, three or 2.5 dimensions. Edges were removed from the graph with a random uniform probability of 0.01 in order to break the symmetry of the lattice.

### 4.2. Roulette wheel selection

A roulette wheel algorithm was implemented similar to [39], where each node corresponds to a particle and a reaction corresponds to a given node being chosen to be fused in the next iteration of the algorithm. Reaction propensities for each of the nodes were set to be proportional to the respective node centrality used (betweenness or closeness) as described in the Results section.

### 4.3. Network-based vasculature generative processes

Two different node centrality measures were used to identify cells for node fusion in the construction of vasculature: BC [45] and CC [43]. BC is calculated as the number of times a node is part of the shortest path that connects a pair of nodes, for all pairs of nodes within a network [45],

$$\text{Betweenness}\;(x)=\underset{i\neq x\neq j}{\sum }\frac{S_{i,j}(x)}{S_{i,j}},$$

where *S_i,j_* is the total number of shortest paths from node *i* to node *j*, and *S_i,j_*(*x*) is the number of those paths that contain the node *x*. The summation is over distinct nodes *i* and *j* that are also distinct from node *x*. Thus, a node is more central the more it is part of paths connecting other pairs of nodes.

Closeness centrality is calculated as the reciprocal of the sum of the length of the shortest paths between the node and all other nodes in the graph [43],

$$\text{Closeness}\;(x)=\frac{n-1}{\sum_{y\neq x}d(x,y)},$$

where *n* is the total number of nodes within the graph and *d*(*x*,*y*) is the distance between nodes *x* and *y*. According to this definition, a node that is closer to other nodes is more central.

Network average path length was calculated using the standard function from NetworkX [56]. This function computes the average shortest distance, in number of discrete jumps, required to reach all the other nodes. This is averaged for all the nodes existing in the graph.

$$\;\mathrm{Average}\;\text{path}\;\text{length}\;(G)=\frac{1}{n(n-1)}\underset{y\neq x}{\sum }d(x,y),$$

where *n* is the number of nodes in the graph *G* and *d*(*x*,*y*) stands for the shortest distance between nodes *x* and *y*. Plots were generated using the standard Python library Seaborn and Matplotlib [57] as well as the NetworkX draw function for the graphs [56].

### 4.4. Global transport efficiency calculation

The vascularized networks were analysed with global efficiency using custom Python code with the calculations described in Latora & Marchiori [58]. Comparisons shown were only made between graphs that started as 992 (31 × 32) nodes for 2D templates, 1000 (10 × 10 × 10) nodes for 3D templates and 972 (18 × 18 × 3) nodes for 2.5D templates before vascularization. The selection of the best solutions in the multi-objective optimization of global efficiency and vasculature fraction was carried out as described in [53],

$$\text{Global}\;\text{efficiency}\;(G)=\frac{1}{n(n-1)}\underset{y\neq x}{\sum }\frac{1}{d(x,y)},$$

where *n* is the number of nodes in the graph *G* and *d*(*x*,*y*) stands for the shortest distance between nodes *x* and *y*.

## Supplementary Material

Figure S1

## Supplementary Material

Figure S2

## Supplementary Material

Figure S3

## Supplementary Material

Figure S4

## Data accessibility

Additional data are available in the electronic supplementary material.
