# Supplementary figures and images for: Efficient vasculature investment in tissues can be determined without global information
Source: J R Soc Interface. 2020 Apr 22;17(165):20200137. doi: 10.1098/rsif.2020.0137 (PMC7211487; doi:10.1098/rsif.2020.0137)

**A**

Average path length

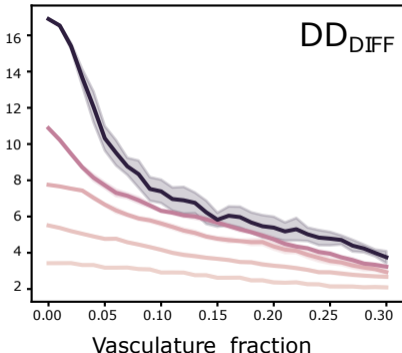**B**

Average path length

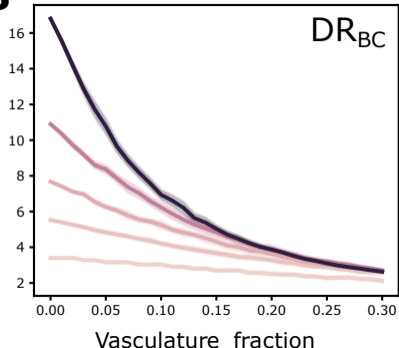**C**

Average path length

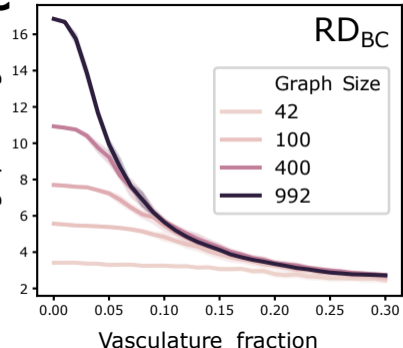

Supplement: Figure S1 [file rsif20200137supp1.pdf]

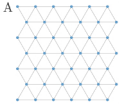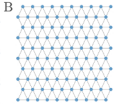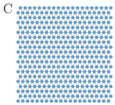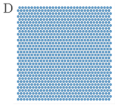

Supplement: Figure S2 [file rsif20200137supp2.pdf]

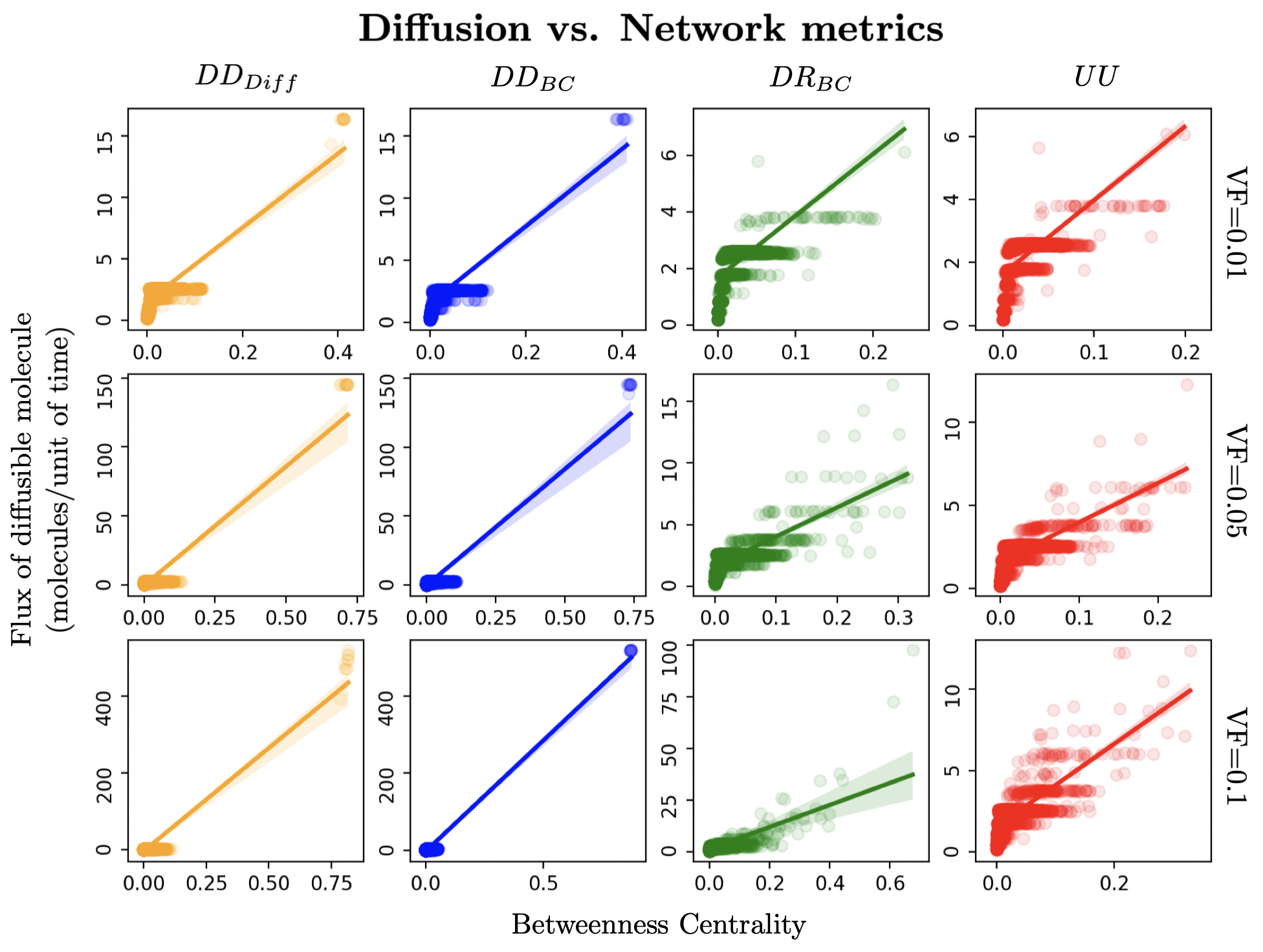

Supplement: Figure S3 [file rsif20200137supp3.png]

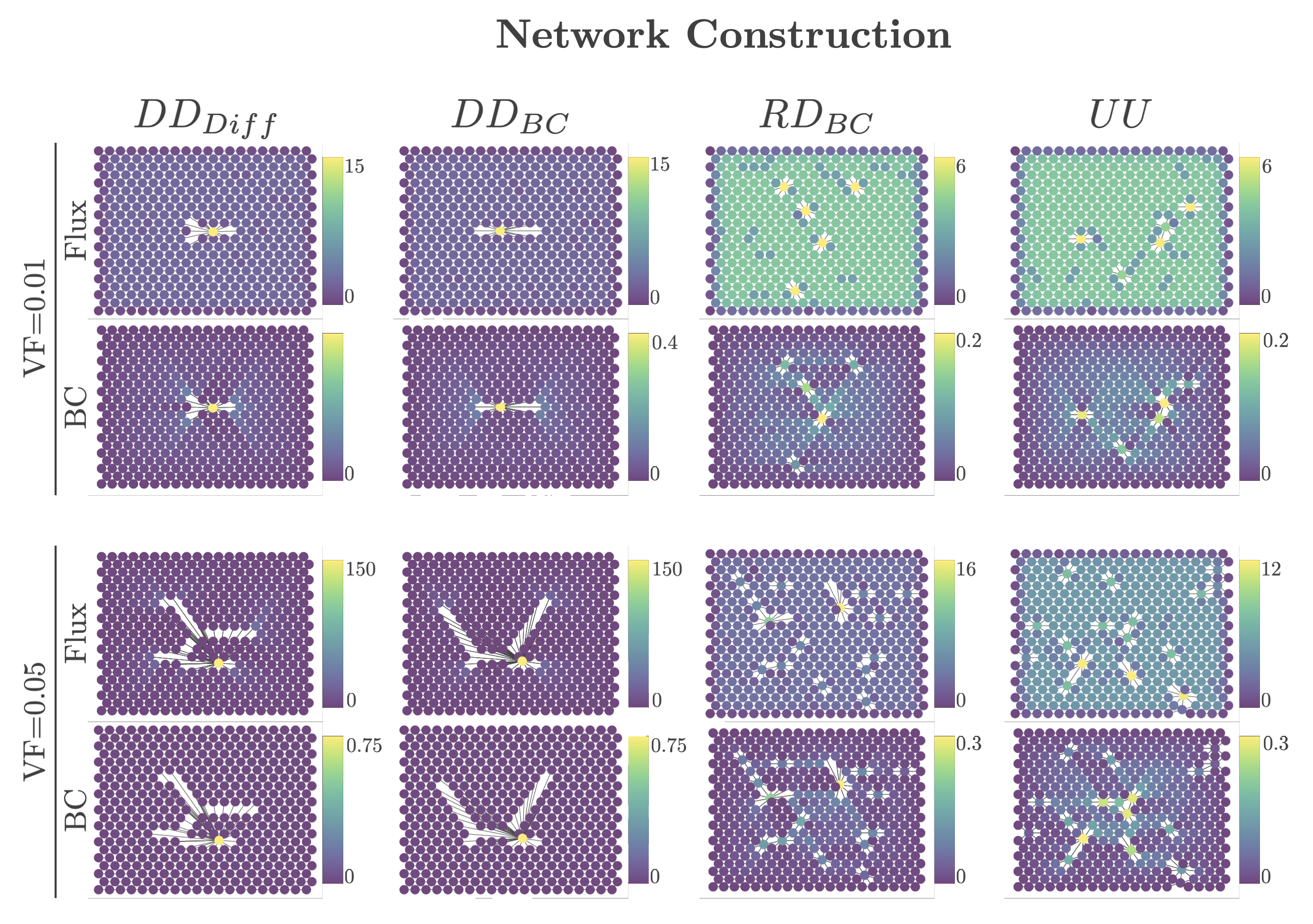

Supplement: Figure S4 [file rsif20200137supp4.png]
